# Supplementary material for: Synthesis and synergistic antibacterial activity of rafoxanide derivative as a novel drug for potentiating colistin activity
Source: Microbiol Spectr. 2026 Mar 30;14(5):e03612-25. doi: 10.1128/spectrum.03612-25 (PMC13141885; doi:10.1128/spectrum.03612-25)
Supplement: Supplemental figures — Fig. S1 to S7. [file spectrum.03612-25-s0001.pdf]

Supplementary material

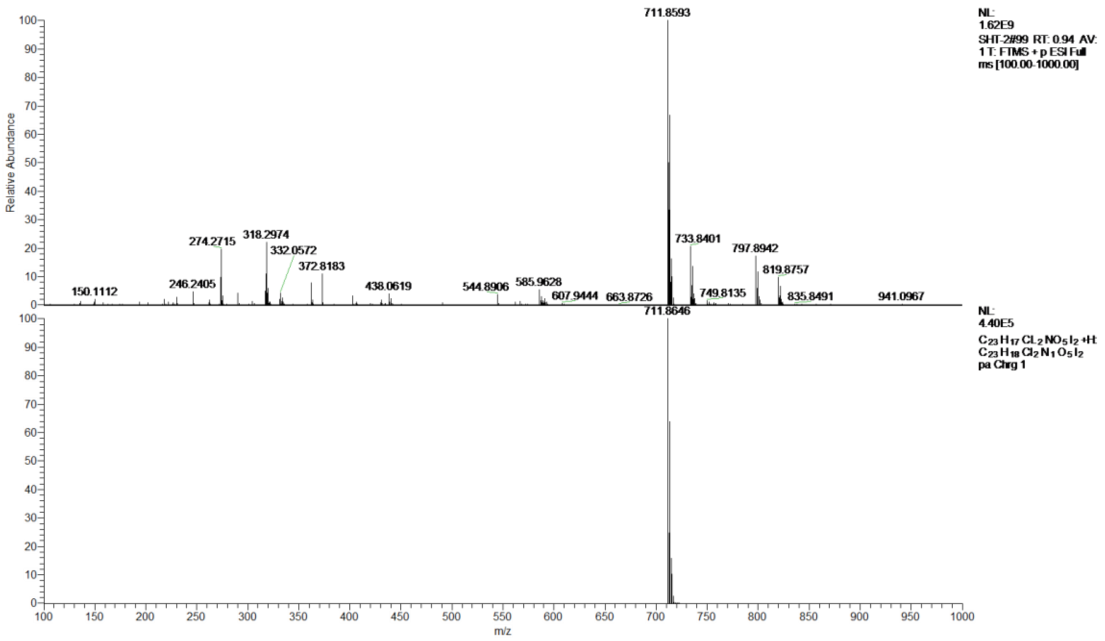

Fig. S1. HRMS spectrum of compound **b** ($C_{23}H_{17}Cl_2I_2NO_5$).

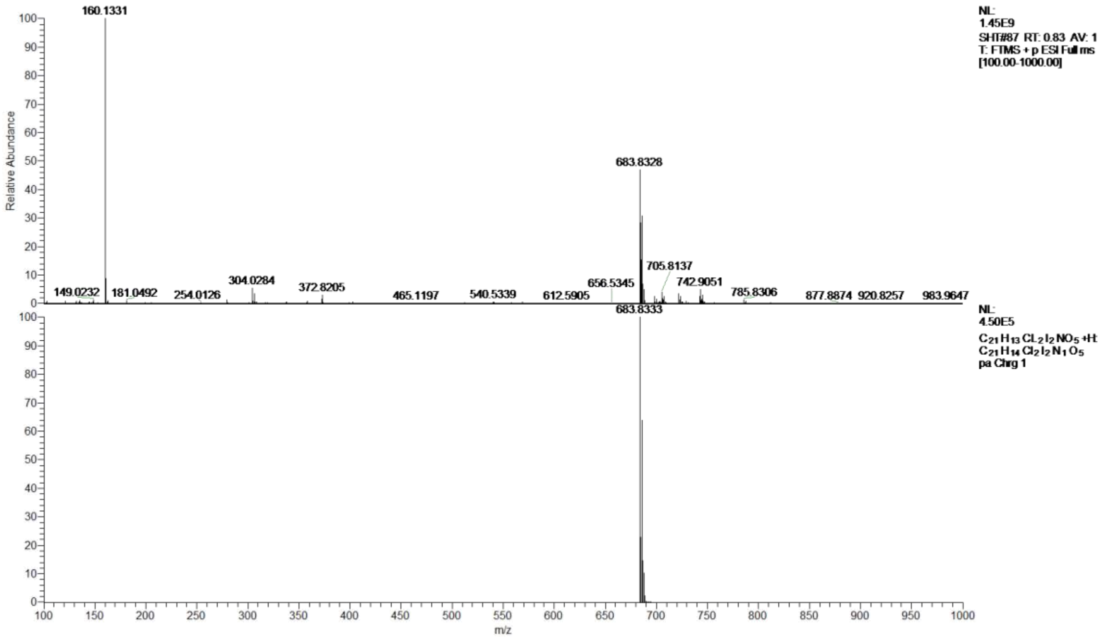

Fig. S2. HRMS spectrum of dikalisuan ($C_{21}H_{13}Cl_2I_2NO_5$).

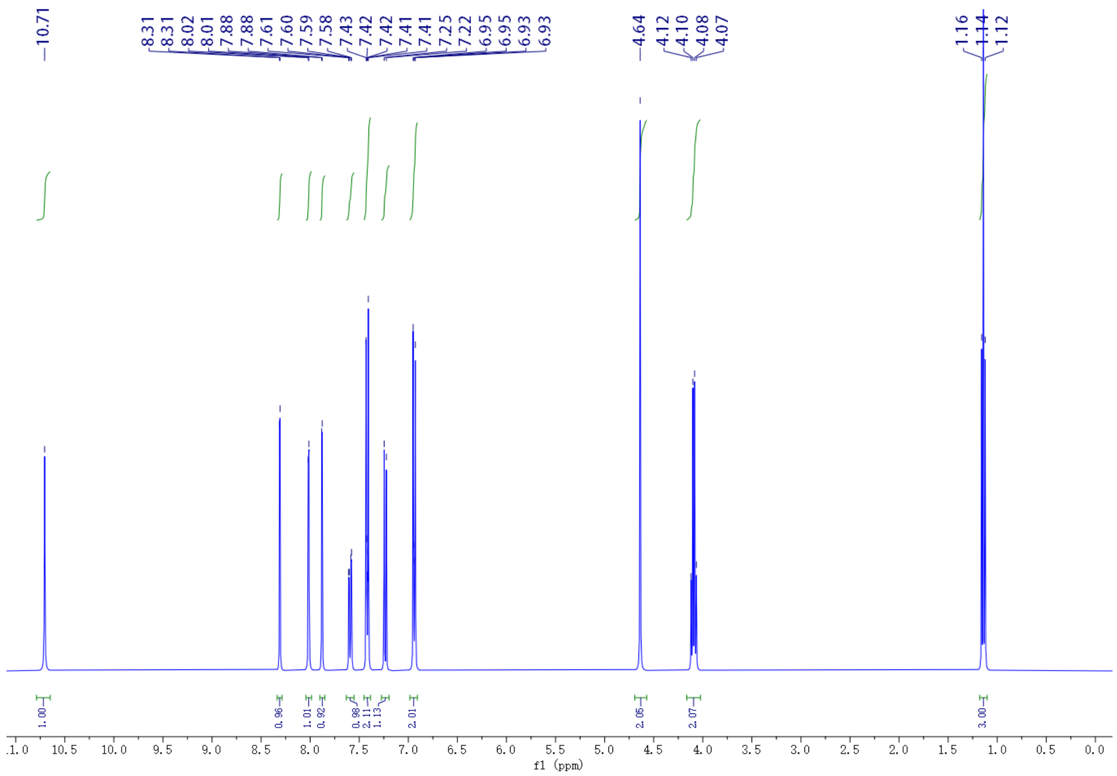

Fig. S3. ^{1}H NMR spectrum of compound **b** ($C_{23}H_{17}Cl_2I_2NO_5$).

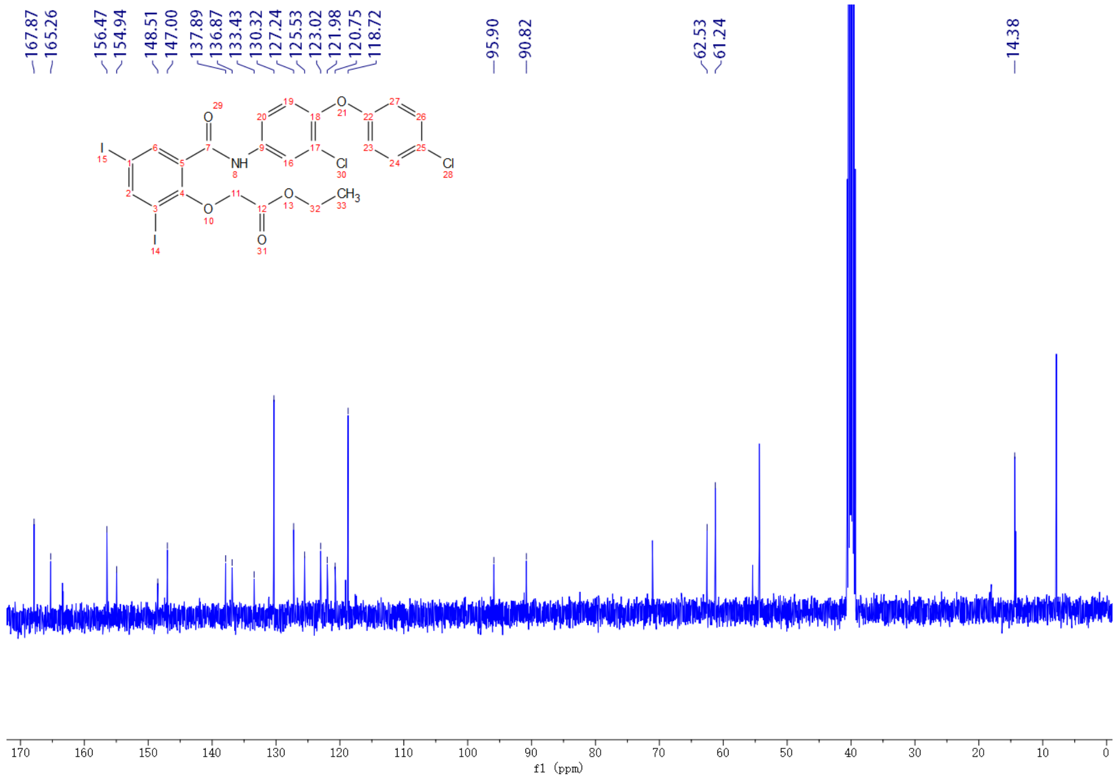

Fig. S4. ^{13}C NMR spectrum of compound **b** ($C_{23}H_{17}Cl_2I_2NO_5$).

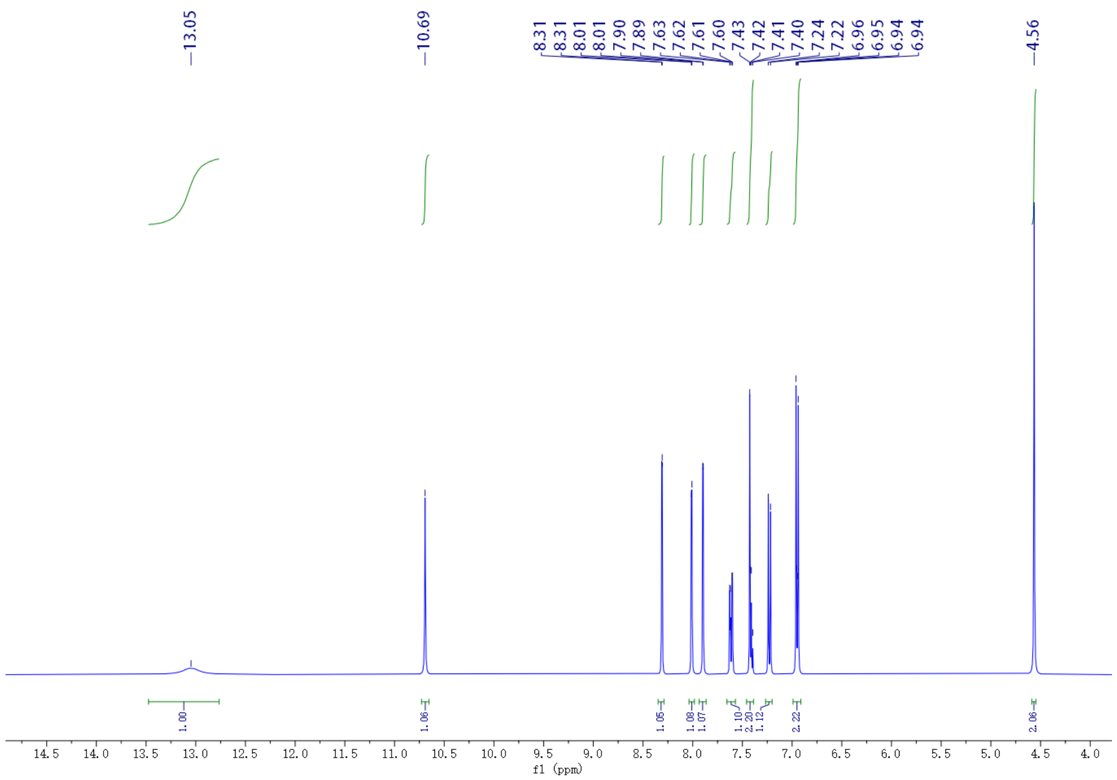

Fig. S5. ^{1}H NMR spectrum of dikalisuan ($C_{21}H_{13}Cl_2I_2NO_5$).

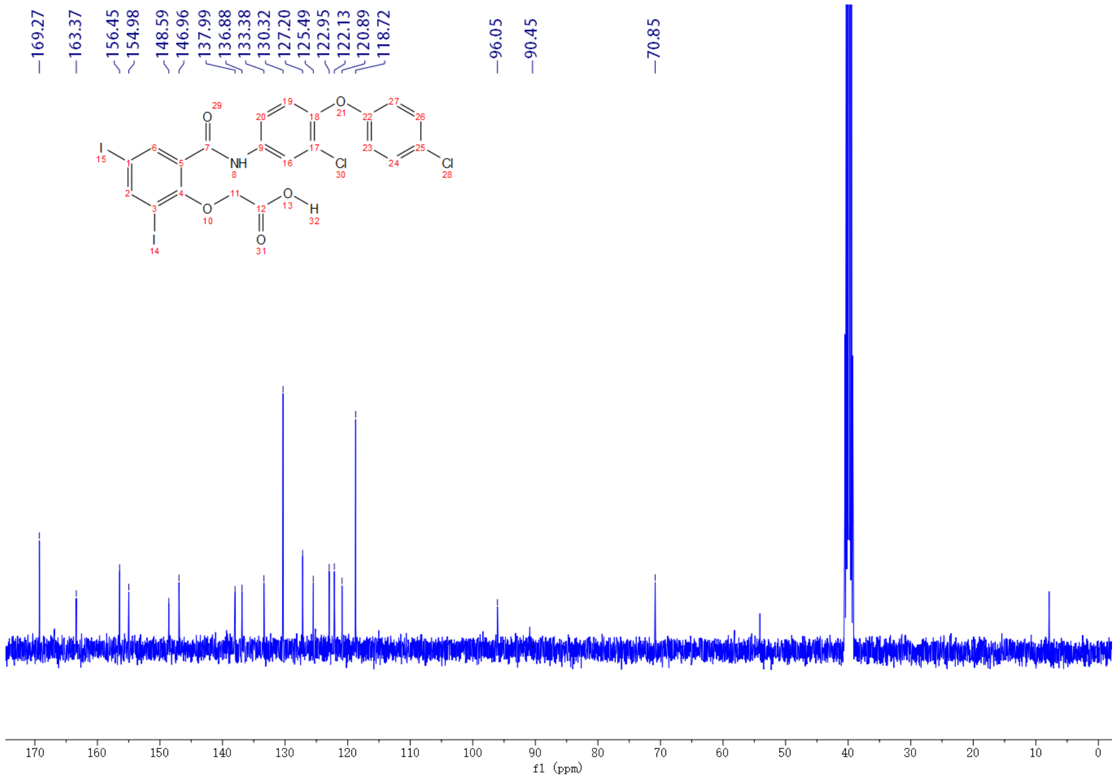

Fig. S6. ^{13}C NMR spectrum of dikalisuan ($C_{21}H_{13}Cl_2I_2NO_5$).

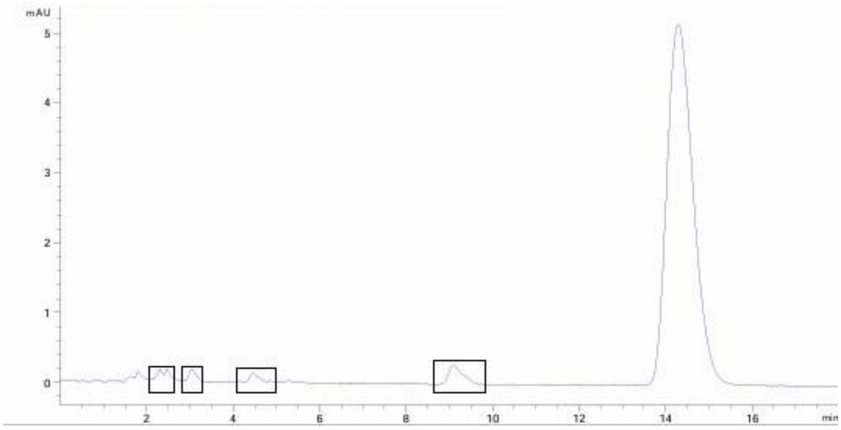

Peak results:

#	[min]		[min]	[mAU*s]	[mAU]	%
1	2.471	MM	0.2218	1.74824	1.31394e-1	0.7760
2	3.047	MM	0.1622	1.60075	1.64466e-1	0.7105
3	4.458	MM	0.1910	1.54877	1.35117e-1	0.6874
4	9.091	MM	0.3208	4.64011	2.41061e-1	2.0595
5	14.289	BB	0.6335	215.76039	5.16315	95.7666

Fig. S7. HPLC detection of dikalisuan ($C_{21}H_{13}Cl_2I_2NO_5$).
